# Role of different recombinant PrP substrates in the diagnostic accuracy of the CSF RT-QuIC assay in Creutzfeldt-Jakob disease

**DOI:** 10.1007/s00441-022-03715-9

**Published:** 2022-12-20

**Authors:** Susana Margarida Da Silva Correia, Matthias Schmitz, Andre Fischer, Peter Hermann, Inga Zerr

**Affiliations:** 1 National Reference Center for TSE and the German Center for Neurodegenerative Diseases (DZNE), Department of Neurology, University Medicine Göttingen, Georg-August University, Robert-Koch-Str. 40, 37075 Göttingen, Germany; 2 Department for Epigenetics and Systems Medicine in Neurodegenerative Diseases, German Center for Neurodegenerative Diseases, 37075 Goettingen, Germany

**Keywords:** Diagnostics, Real-time quaking-induced conversion, Creutzfeldt-Jakob disease, Recombinant prion protein substrates

## Abstract

The development of the real-time quaking-induced conversion (RT-QuIC), an in vitro protein misfolding amplification assay, was an innovation in the scientific field of protein misfolding diseases. In prion diseases, these types of assays imitate the pathological conversion of the cellular prion protein (PrP^C^) into a protease-resistant and/or amyloid form of PrP, called PrP resistant (PrP^Res^). The RT-QuIC is an automatic assay system based on real-time measuring of thioflavin-T (Th-T) incorporation into amyloid fibrils using shaking for disaggregation. It has already been applied in diagnostics, drug pre-screening, and to distinguish between different prion strains. The seeded conversion efficiency and the diagnostic accuracy of the RT-QuIC assay strongly depend on the kind of recombinant PrP (rec PrP) substrate. The DNA sequences of different substrates may originate from different species, such as human, bank vole, and hamster, or from a combination of two species, e.g., hamster-sheep chimera. In routine use, either full-length (FL) or truncated substrates are applied which can accelerate the conversion reaction, e.g., to a more sensitive version of RT-QuIC assay. In the present review, we provide an overview on the different types of PrP substrates (FL and truncated forms), recapitulate the production and purification process of different rec PrP substrates, and discuss the diagnostic value of CSF RT-QuIC in human prion disease diagnostics.

## Principle of the RT-QuIC

Prion diseases, such as Creutzfeldt-Jakob disease (CJD), fatal familial insomnia (FFI), or Gerstmann-Sträussler-Scheinker syndrome (GSS), exhibit a template-induced conversion of the cellular prion protein (PrP^C^) into an abnormally folded isoform, termed PrP scrapie (PrP^Sc^). Protein misfolding amplification assays mimic the seeded conversion process of PrP in vitro to amplify minuscule amounts of a PrP^Sc^ seed to an aggregated form of proteinase K–resistant PrP, termed (PrP^Res^) (Atarashi et al. 2011, 2008; Saborio et al. 2001). Transmission studies had indicated that this aggregated form of PrP does not always induce a prion disease. Depending on the kind of tissue and the substrate sequence used for amplification, it is considered to be different from the original transmissible PrP^Sc^ seed (Raymond et al. 2020).

The first protein misfolding aggregation assays, such a protein misfolding cyclic amplification assays (PMCA), were based on sonication and applied non-infected brain material as source of PrP^C^ substrate (Saborio et al. 2001). The replacement of sonication by quaking, the addition of thioflavin-T (Th-T) (incorporates into growing amyloids), and the application of bacterially synthesized rec PrP substrate were further improvements of the next generation of prion seeding amplification assays, called real-time quaking-induced conversion (RT-QuIC) assay (Atarashi et al. 2011; Wilham et al. 2010; Orrú et al. 2015a). Later, a further development was the enhanced-QuIC (eQuIC). Due to an additional pre-analytical immunoprecipitation step of PrP, the eQuIC for blood or brain is more sensitive than the standard RT-QuIC (Orrú et al. 2011). This additional step is more time-consuming and costly and would not make sense for CSF diagnostic. Consequently, the majority of diagnostic laboratories currently apply the standard RT-QuIC as a routine test for prion disease diagnostics.

Compared to the PMCA, the RT-QuIC assay facilitates the standardization of PrP^Sc^ amplification and detection which is a prerequisite for routine use.

In the RT-QuIC reaction, small amounts of a misfolded PrP^Sc^ seed which can be derived from the brain, cerebrospinal fluid (CSF), olfactory mucosa (OM) brushings, or other tissues from CJD patients (Atarashi et al. 2011; Orrú et al. 2015a, 2014) may bind and convert rec PrP substrate molecules by changing their conformation and integrating them in an amyloid aggregate. During the conversion process, rec PrP substrate molecules achieved a seeding-competent state which was firstly shown by *Atarashi *et al*.* for CSF (Atarashi et al. 2011).

During the RT-QuIC, samples are subjected to cycles consisting of incubation and vigorous shaking. The shaking causes a fragmentation of PrP^Res^ aggregates into smaller conversion-competent seeds. After a lag phase, the RT-QuIC product, containing β-sheet structures, may interact with the fluorescent Th-T dye resulting in an increase of fluorescence signal. The seeded conversion process and the sigmoidal signal increase can be monitored in real-time using a fluorescence plate reader.

To evaluate the seeded conversion efficiency, the duration of the lag phase, area under the curve (AUC), and maximal signal intensity can be used as semi-quantitative parameters to compare the seeding conversion efficiencies of different groups (Cramm et al. 2015, 2016; Schmitz et al. 2016a). The 96-well plate format of the RT-QuIC enables the automatic analysis of multiple reactions (of more than 30 different samples in triplicates) facilitating the use of RT-QuIC in routine diagnostics.

## Production of recombinant PrP substrates

**Fig. 1:**
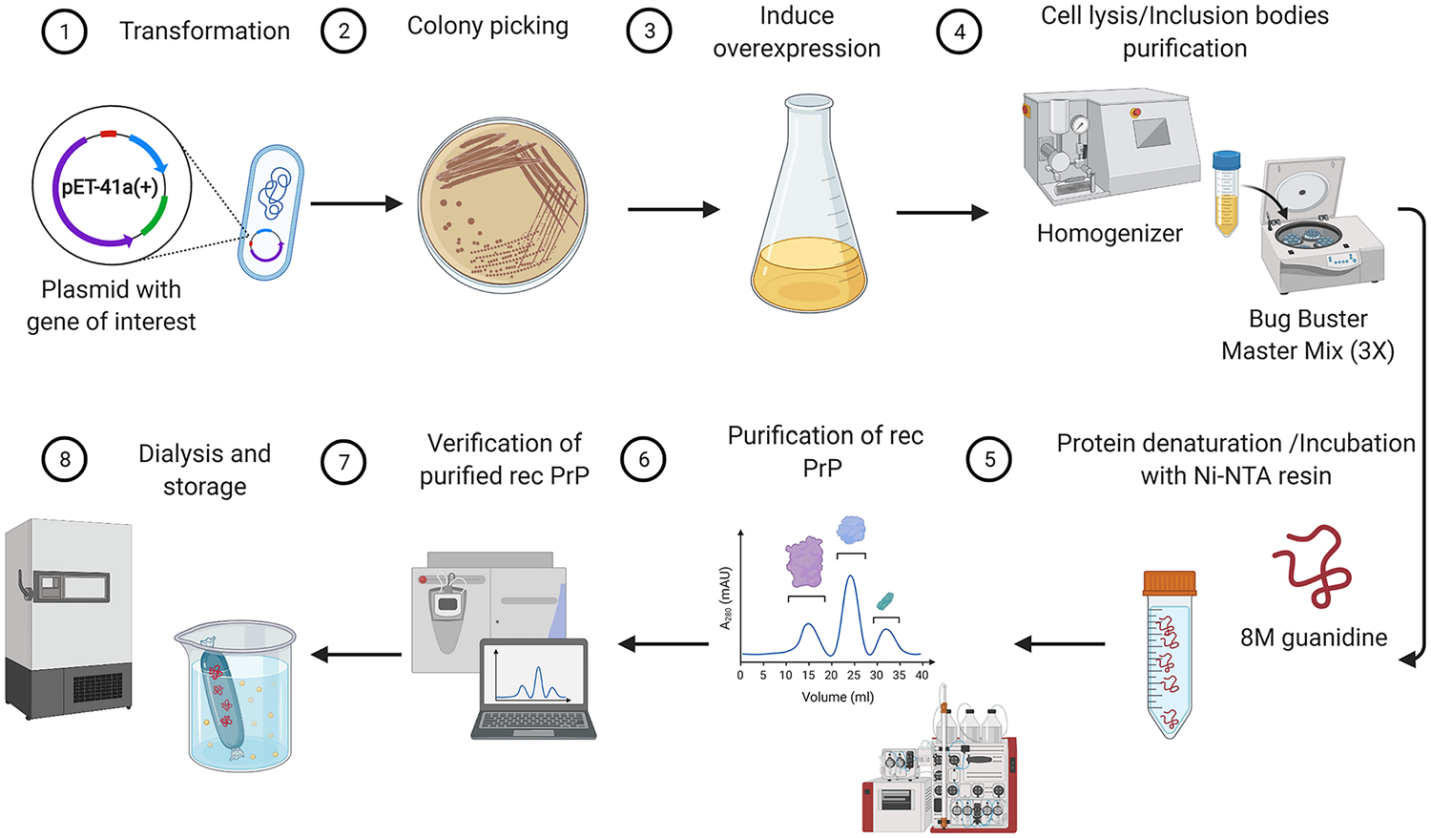
Overview on different steps of rec PrP production and purification. The first step in protein purification is the insertion of the vector (e.g., pET-41a( +)) into competent *E. coli* bacteria via heat shock transformation (**1**). The transformed bacteria are plated on LB agar plates with selective antibiotics (**2**). Due to the vector-induced antibiotic resistance, only bacteria with vector will grow (**2**). A single colony is picked from the agar plates and incubated in LB medium for overexpression by IPTG induction or overnight in auto-induction medium (**3**). Subsequently, bacteria will be centrifuged, the pellet will be weighted, and the BugBuster Master mix or lysis buffer will be added according to the pellet weight followed by homogenization and centrifugation (**4**). Once the inclusion bodies are purified, the pellet becomes denatured and incubated with Ni–NTA beads (**5**). After column is attached to ÄKTA pure micro system, rec PrP becomes refolded and eluted (**6**). The purified rec PrP is collected from the elution step and verified either by Western blotting, Coomassie staining, and/or mass spectrometry (**7**). The last step in protein purification is the dialysis of the purified rec PrP followed by storage at − 80 °C (**8**) (created by BioRender)

Multiple protein expression systems are available for the production of recombinant proteins, e.g., bacteria, yeast, filamentous fungi, and unicellular algae. However, *Escherichia coli* is the host system widely used in the production of the rec PrP due to multiple significant benefits over other expression systems including a well-established protein overexpression protocol, low costs, ease of use, and scale (Terpe 2006). A vast number of *Escherichia coli* strains as well as plasmid vectors with a variety of choices of promoters, affinity tags, and antibiotic resistance are commercially available with the purpose to confer the best results of transformation in the *Escherichia coli* and an ultimate yield in protein expression. Due to the intrinsic affinity of the PrP^C^ for metals, the additional His-tag sequence is not needed as fusion to PrP to obtain a simple and faster protein purification (Csire et al. 2020; Jackson et al. 2001; Schmitz et al. 2016a). PrP expression in *Escherichia coli* can be achieved overnight by using auto-induction media or by IPTG protocols (Fig. 1). Commercial or homemade auto-induction media have preferentially been used for the expression of recombinant proteins due to being convenient since the bacterial growth does not need to be measured and due to a higher yield of protein (several-fold higher than conventional IPTG induction systems) (Grabski et al. 2005; Studier 2005). The majority of rec PrP is localized in the inclusion bodies. The isolation of these inclusion bodies can be achieved by a purification protocol for inclusion bodies (Palmer and Wingfield 2004) or with the use of BugBuster Master Mix buffer, which so far is the most effective and fastest method (Fig. 1) (Bourkas et al. 2019). It allows a maximal recovery of functional proteins from Gram-negative and Gram-positive bacteria. After purification, inclusion bodies are denatured (by guanidine or urea buffer) with the purpose to release the overexpressed proteins in the supernatant.

Protocols using urea as a denaturing agent are more time-consuming in the purification process than guanidine-based protocols (Makarava et al. 2017; Wilham et al. 2010) due to the gradual spontaneous conversion of urea in an aqueous solution to ammonium cyanate (Mooshammer et al. 2020). This needs to be removed by ion-exchange chromatography due to the potential reaction with protein amino groups and the induction carbamylation (Kollipara and Zahedi 2013; Pietrement et al. 2013). After denaturation of inclusion bodies, the supernatant is incubated in the nickel resin allowing the binding of the denatured proteins to the beads (Fig.1). The refolding and elution steps can be run either in a gradient or linear program.

In a linear gradient program, the refolding buffer passes the column without mixing with previous equilibration buffers. This program is faster than the gradient program but may lead to inappropriate refolding of proteins. When used a gradient refolding protocol, a linear refolding gradient could be run prior to a gradient elution to ensure a successful refolding.

After elution, proteins in the center of the large peak are collected and mixed with 1/3 of dialysis buffer to equilibrate the proteins to the pH and filtered to avoid possible aggregation. To remove imidazole, purified rec PrP (0.4–0.6 mg/mL) is subjected to dialysis and to filtration (0.2-micron filter) (Wilham et al. 2010). After dialysis, rec PrP needs to be verified either by Western blotting, Coomassie staining, and/or mass spectrometry (Fig. 1). Finally, rec PrP will be subjected to a functional validation using retrospective well-classified samples in order to exclude a potential self-aggregation resulting in unspecific false-positive RT-QuIC signals.

## Influence of distinct rec PrP substrates on the seeded conversion reaction and the diagnostic accuracy of the RT-QuIC

The diagnostic criteria for CJD are based on clinical characteristics, MRI, and electroencephalographic measurements as well as the detection of surrogate CSF biomarkers, such as the proteins 14–3-3 and tau (Llorens et al. 2017; Schmitz et al. 2016b; Zerr et al. 2009). A confirmation of a prion disease diagnosis requires a direct detection of the pathogenic PrP^Sc^ by immunoblot and immunohistochemical staining upon autopsy (WHO 2003). In this context, in vitro protein misfolding amplification assays, such as the RT-QuIC, have been well established in the last years and allow a reliable direct detection of PrP^Sc^ in biological fluids of prion disease patients. A relevant compound in the RT-QuIC assay across different reference laboratories is the kind of rec PrP substrate used. The rec PrP sequences are from different species, such as human (Atarashi et al. 2011), hamster (McGuire et al. 2012), and bank vole (Orrú et al. 2015b), or from two species, such as the hamster-sheep chimera, and show different diagnostic accuracies (Table 1). Atarashi et al*.*, who firstly reported the application of CSF RT-QuIC in prion disease diagnostics using rec PrP with the full-length (FL) human sequence, reported a sensitivity of more than 80% and a specificity of 100% in a cohort of 18 CJD patients and 30 controls (Atarashi et al. 2011). Subsequently, a study, executed by *McGuire *et al., applied FL hamster rec PrP and confirmed its very high specificity of 99% and a sensitivity of 89% in an exploratory cohort of 123 sCJD patients and 103 control cases (McGuire et al. 2012). Another study with FL hamster PrP substrate conducted in a larger cohort confirmed the good diagnostic accuracy of FL hamster PrP substrate (88% sensitivity and 99% specificity) (Lattanzio et al. 2017) (Table 1).

**Table 1:** Overview on the diagnostic accuracy of the CSF RT-QuIC in different studies

Species	Amino acid res	Accuracy for sCJD CSF diagnostics Sensitivity / Specificity		References
Human	23–231	> 80% (27/34)	100% (0/49)	Atarashi et al. 2011
Hamster	23–231	89% (109/123) 88.2% (148/179)	99% (1/103) 99.4% (1/163)	McGuire et al. 2012 Lattanzio et al. 2017
	90–231	94% (106/113) 92% (102/111) 95% (60/63)	100% (0/64) 98.5% (1/67) 100% (14/14)	Orrú et al. 2015a; Groveman et al. 2016 Foutz et al. 2017 Foutz et al. 2017
	90-231	96% (58/61)	100% (0/80)	Fiorini et al. 2020
Bank vole	23-230	88.6% (70/79)	91.2% (5/57)	Orrú et al. 2015b; Mok et al. 2021
Hamster-sheep chimera	Hamster 23–137 Sheep 141–234	80% (51/64) 89% (163/183)	99% (2/400) 100% (0/118)	Cramm et al. 2015, 2016 Hermann et al. 2018

Another study from 2015 applied a chimeric form of rec PrP consisting of a hamster-sheep sequence, which is less prone to self-aggregation and which enables the differentiation between different types of PrP^Sc^ in familial CJD (E200K), FFI and sCJD patients (Cramm et al. 2015). The hamster-sheep chimera was reported with a sensitivity of 80–85% and a specificity of 99% for CJD (Cramm et al. 2016). A more recent study calculated for FL bank vole rec PrP a sensitivity of 88.6% which is comparable to other studies; however, the specificity (91%) remained lower (Mok et al. 2021) (Table 1). The advantage of bank vole rec PrP compared to other substrates is the amplification and detection of many different PrP^Sc^ strains making it interesting for the diagnostics of different types of prion diseases and subtypes of sCJD (McGuire et al. 2016; Orrú et al. 2015b). For example, the sensitivity of RT-QuIC (in particular the hamster-sheep substrate) is relatively low for the diagnostics of some genetic prion diseases, such as FFI or GSS, compared to other markers (Schmitz et al. 2022). For these types of prion diseases, the use of bank vole substrate may be beneficial.

Besides the standard or first generation of RT-QuIC, mainly applying FL rec PrP, more recent studies reported a second generation of the RT-QuIC. Instead of FL PrP, they applied a truncated hamster rec PrP substrate (amino acids 90–231) combined with some modification of the RT-QuIC protocols (incubation at 55 °C and 0.002% sodium dodecyl sulfate (SDS) addition) (Orrú et al. 2015a; Foutz et al. 2017; Franceschini et al. 2017; Groveman et al. 2017). The use of a truncated form of rec PrP accelerated the seeded conversion efficiency of the RT-QuIC, indicated by a shorter lag phase of less than 20 h, and the total running of the assay was significantly reduced (Foutz et al. 2017; Franceschini et al. 2017; Groveman et al. 2017). Additionally, a sensitivity of 94% (113 CJD samples) without loss of specificity (100%) was obtained (Foutz et al. 2017; Franceschini et al. 2017; Groveman et al. 2017; Thompson and Mead 2019).

Comparing the diagnostic accuracies of all rec PrP substrates used in the RT-QuIC for CJD, the sensitivity varies between 80 and 96%, whereas most studies observed specificities between 99 and 100%. This consistently excellent specificity is the unique feature of the RT-QuIC compared to surrogate biomarkers, such as 14–3-3, tau, or alpha synuclein. Beside the use of different rec PrP substrates, the variability of the CSF RT-QuIC sensitivity in CJD diagnosis may depend on different (pre)-analytical protocols. In addition, the composition of patient cohorts, such as the controls (healthy, neurological without neurodegeneration, or neurological with neurodegeneration) or case group characteristics (e.g., different sCJD disease subtypes, disease stage, and the use of cases with clinical or neuropathological diagnosis), may contribute to variable accuracies. To calculate the diagnostic accuracy, many studies applied CJD samples, classified as probable or possible CJD, because confirmation upon autopsy is often missing. Misdiagnosed CJD cases may interfere with the diagnostic accuracy of the assay. A further cause for variability is the kind of (pre)-analytic protocol. Regarding the CSF RT-QuIC (routinely applied in diagnostics), a variety of instruments, rec PrP substrates, and CSF volumes are used across different CJD surveillance centers. Most of them apply hamster rec PrP, either as FL or as truncated form (rec PrP 90–231), exhibiting a very good diagnostic accuracy (Table 1). However, to find the best suitable substrate with the best diagnostic accuracy would require a direct comparison of different rec PrP substrates in the same laboratory and under the same (pre)-analytical conditions, such as the same RT-QuIC protocol, patient cohorts, and same experimenter.

Therefore, we propose that more than one rec PrP substrate is appropriate for a reliable detection of PrP^Sc^ in CSF of CJD patients. Ring trial studies had proven an excellent reproducibility of CSF RT-QuIC across the European Creutzfeldt-Jakob Disease Surveillance Network (McKenzie et al. 2022). Long-term experiences in handling, the kind of prion disease (sCJD, FFI, GSS), and the availability might be the decisive criterion for substrate selection across different reference laboratories.

## Conclusion

Several rec PrP substrates of different length (FL vs. truncated forms) and including various sequences from different species have been successfully applied in the RT-QuIC to detect PrP^Sc^ in human prion diseases. The diagnostic accuracy may vary between 80 and 96% sensitivity and 91 and 100% specificity depending on the type of substrate, the applied protocol, and the patient cohort. In particular, the high specificity of the RT-QuIC is an advantage over other body fluid biomarkers for neurodegenerative diseases. Ring trials among reference laboratories have proven the high reproducibility of the RT-QuIC; however, the unification of the used rec PrP substrate may result in a further improvement. The selection of the most suitable substrate for the RT-QuIC may depend on different factors, such as the kind of body fluid or tissue, the type of prion strain, the desired specificity of almost 100%, or the time of the assay duration.

## Data Availability

Available underlying data will be provided upon reasonable request.
